# Analytical Approach for Forecasting the Load Capacity of the EN AW-7075-T6 Aluminum Alloy Joints Created Using RFSSW Technology

**DOI:** 10.3390/ma17071529

**Published:** 2024-03-27

**Authors:** Rafał Kluz, Magdalena Bucior, Andrzej Kubit, Tomasz Trzepieciński, Katarzyna Antosz, Koen Faes

**Affiliations:** 1 Department of Manufacturing and Production Engineering, Rzeszow University of Technology, Al. Powst. Warszawy 8, 35-959 Rzeszow, Poland; rkluz@prz.edu.pl (R.K.); magdabucior@prz.edu.pl (M.B.); akubit@prz.edu.pl (A.K.); tomtrz@prz.edu.pl (T.T.); 2 Belgian Welding Institute, Technologiepark Zwijnaarde 935, B-9052 Ghent, Belgium; koen.faes@bil-ibs.be

**Keywords:** aluminum alloy, artificial neural networks, optimization, RFSSW

## Abstract

To ensure the high reliability of aircraft structures, the Refill Friction Stir Spot Welding (RFSSW) process must be characterized by a high load capacity of the welds and a small standard deviation of the load capacity spread. This allows us to obtain uniform functional properties in the connections, ensuring the high quality of the process. This work aims to select the most favorable technological parameters for the welding process of EN AW-7075-T6 Alclad aluminum alloy sheets, which are used for the production of aircraft structures. The best networks were calculated using the Statistica 13.3 program. The obtained results were compared with the results of previous investigations. It has been shown that a model using neural networks allows for the determination of connection parameters with much greater accuracy than the classical model. The maximum error in estimating the load capacity of the connection for the mathematical model was 6.13%, and the standard deviation was 14.51%. In the case of neural networks, the maximum error value did not exceed 1.55%, and the standard deviation was 3.74%. It was shown that, based on the neural model, it is possible to determine the process parameters that ensure the required quality capacity of the process, ensuring a probability of obtaining the required load capacity of the connections amounting to P = 0.999935 with a defect rate of 0.0065%. This possibility is not provided by the classical model due to its large error in estimating the process spread and the high sensitivity of the process input parameters to the output parameters.

## 1. Introduction

**Figure 1:**
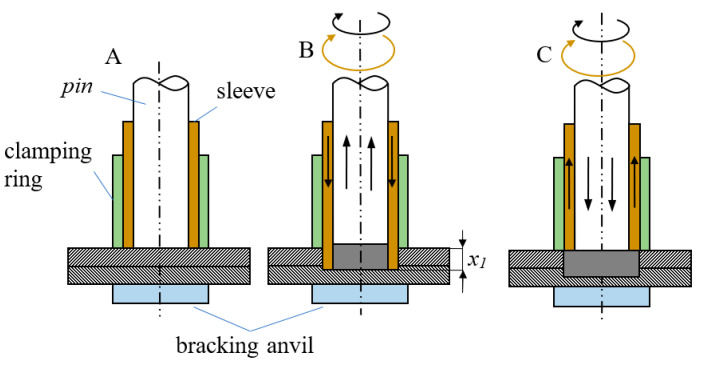
RFSSW welding process: (**A**) plasticization of the material, (**B**) recess of the sleeves, and (**C**) refill.

Refill friction stir spot welding (RFSSW) is a modern process for joining sheet metal in a solid state. In industry, friction welding replaces other joining methods, such as riveting or gluing. The undoubted advantage of RFSSW is that it does not weaken the structure, reduces the time-consuming assembly process, and, in the case of aircraft structures, improves their aerodynamics [1,2]. The welding process is performed in several phases [3,4,5]. In the first stage (Figure 1A), the pin and sleeve are pressed against the joined materials, immobilizing them. The simultaneous rotation and pressure of the pin and sleeve heat the material and cause it to plasticize. In the second stage of the process (Figure 1B), the sleeve penetrates into the material and the pin retracts, which provides space for the material to be moved. In the last phase (Figure 1C), the counter-rotating movement of the pin and the sleeve takes place, which squeezes the plasticized metal toward the joint. At the end of the process, the sleeve and pin return to their original position, and the tool is moved away from the weld.

However, issues related to the processes occurring during welding are not fully known, which causes difficulties in selecting the optimal process conditions for specific aircraft alloys. The authors of many works investigated the impact of the parameters of the process on the joint load capacity (LC), obtaining varying results both in terms of the significance of the impact of individual parameters and their optimal values. Zhaohua et al. [6] conducted research on spot welding of 1 mm-thick 5052-H112 aluminum alloy sheets, showing that the welding time has no significant impact on the joint load capacity. In turn, research on the welding process of AA3003H11 aluminum alloy sheets conducted by Mumin et al. [7] showed that all parameters of the process, that is, rotational speed, tool depth, and welding time, have a dominant impact on the process. Shen et al. [8] investigated the welding process of Al6022-T4/Al7075-T6 sheets, showing that the strength of the joints increases with increasing welding time and tool recess depth. They also demonstrated the significant influence of tool shape on the shear strength of the joint. Camila et al. [9] examined the impact of the tool wear process on the LC of joints made of AA2198-T8 sheet metal. They determined that the main mechanism of tool failure affecting the weld structure is adhesive and plastic deformation. As a result of the tests, a tendency to reduce the shear strength of the welds as a function of the wear of the tool was shown.

Feizollahi and Moghadam [10] studied the process of joining 6061-T6 aluminum sheets with low carbon galvanized steel. During the research, they used three types of tools. As a result of their research, they showed that the use of short conical pins in the welding process allows one to increase the microhardness of the joint in the aluminum plate, while increasing it leads to an increase in the microhardness of the joint in the steel plate. They showed that the most important parameter of the welding process is the depth of penetration of the tool and the welding time. Zou et al. [11] investigated the process of joining 2 mm-thick 2219-O aluminum sheets with 2219-C10S plates of different thicknesses (from 4 mm to 14 mm). The tests showed that as the thickness of the bottom plate increases, the maximum joint temperature decreases. Tensile and compressive stresses were also shown to be distributed mainly along the force direction of the weld nucleus. However, shear stresses mainly occured in the direction perpendicular to the acting force. Moreover, bending stresses were also found in the connections, with their value depending on the thickness of the bottom sheet. Yangfan et al. [12] proposed a novel spot welding technique using large tools. As a result of their research, they showed that the tool recess has a significant impact on the type of joint damage and the way the joints break. Tiwan et al. [13] investigated the effect of tool rotational speed and pin geometry on the structure and mechanical strength of the AA2024-O/AA6061-T6 joints. They showed that increasing the rotational speed of the pin causes an increase in frictional heat, which leads to grain coarsening. In all analyzed zones, the hardness values of the upper AA2024-O sheet are higher than those of the parent metal due to precipitation hardening during welding, while the lower AA6061-T6 sheet softened in the weld area and adjacent area. At low tool speeds, the weld exhibits shear failure. As the rotational speed increases, the method of joint destruction changes to mixed.

Modern modeling techniques based on neural networks and the support vector method are increasingly used to solve complex engineering problems, which can potentially provide better prediction accuracy. Wang et al. [14], based on a neural model using non-dominated sorting genetic algorithm II (NSGA II), solved the problem of multi-criteria optimization in the grinding process. They presented compromise solutions between criteria related to energy and time efficiency and product quality. Li et al. [15] proposed a method to solve the problem of ensuring the surface quality of grinding products by monitoring and recording force signals in real time. Based on the Improved Fireworks Algorithm (IFWA), they determined the functional relationship between the grinding force signal and the amplitude–frequency curve of the surface texture. Zhao et al. [16] developed a surface roughness prediction model algorithm based on ensemble learning using support vector machines (ELSVM), which can be used to predict the surface roughness of LUVAG alumina ceramics. They compared the developed method with machine learning methods. They showed that the ELSVM model has the lowest average mean absolute error (MAE) for surface roughness prediction. Li et al. [17] proposed an adaptive support vector machine (ADSVM) model by integrating adaptive sampling technology (AST) and the developed directed support vector machine (DSVM) to improve the efficiency and accuracy of reliability analysis of aircraft engine turbine blades. They showed that the model (ADSVM) is characterized by higher accuracy and efficiency compared to the classical method of determining reliability by 20% and 46.2%, respectively.

The load capacity of friction-welded joints made of aluminum alloy depends on many parameters. Structure inhomogeneities often appear in the weld core due to insufficient mixing of the materials of the joined sheets. In the aviation industry, sheets are additionally protected against corrosion by a coating (Alclad) with properties that differ significantly from the properties of the sheet material. This causes uneven heat flow in the weld area, causing additional joint defects. Therefore, the precise determination of the process parameters is a very time-consuming task and requires many experimental tests. Classical methods of conducting and processing research results also have significant limitations, and the results obtained on their basis may be precise enough. Therefore, the aim of the article was to develop a model that presents the influence of the technological parameters of the RFSSW process using artificial neural networks, which allow one to take into account many more factors in the learning process than classical models. This enables a more accurate prediction of connection load capacity and the quality of the RFSSW process.

## 2. Material and Methods

**Figure 2:**
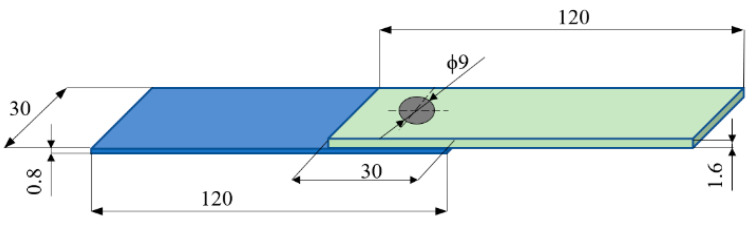
Test specimen.

The test specimens were made on the RPS 100 VA11 welding device manufactured by Harms & Wende GmbH and Co KG (Hamburg, Germany). Sheets of EN AW-7075-T6 [18] aluminum alloy (AMAG Rolling Gmbh, Ranshofen, Austria) were welded with a thickness of 0.8 mm and 1.6 mm, with an overlap of 30 mm in length and width (Figure 2). The research presented in this work is part of broader research aimed at examining the properties and selecting optimal welding parameters for joints used in thin-walled structures. The aim of the research is to demonstrate the possibility of using RFSSW technology as an alternative to riveting, which is typically used in such structures. Specifically, this concerns the structure of aircraft fuselage, in which the skin sheet is stiffened by longitudinal stringers and transverse frames. These elements are made of typical aircraft aluminum alloys, such as the EN AW-7075-T6 aluminum alloy used. The selection of sample geometry resulted directly from the geometry of the skin–stringer joint. Hence, 0.8 mm-thick sheets corresponding to the skin are joined with 1.6 mm-thick sheets corresponding to the stringers. The joints are made in rows, maintaining a spacing between spots of approximately 30 mm. This spacing determines the width of the overlap. Since for this type of spot joint, according to most standards, the length of the overlap is equal to its width, the dimension of 30 mm × 30 mm was adopted. The tests were carried out on materials provided by a certified supplier of production materials in the aviation industry. Materials from one batch were used for testing.

**Figure 3:**
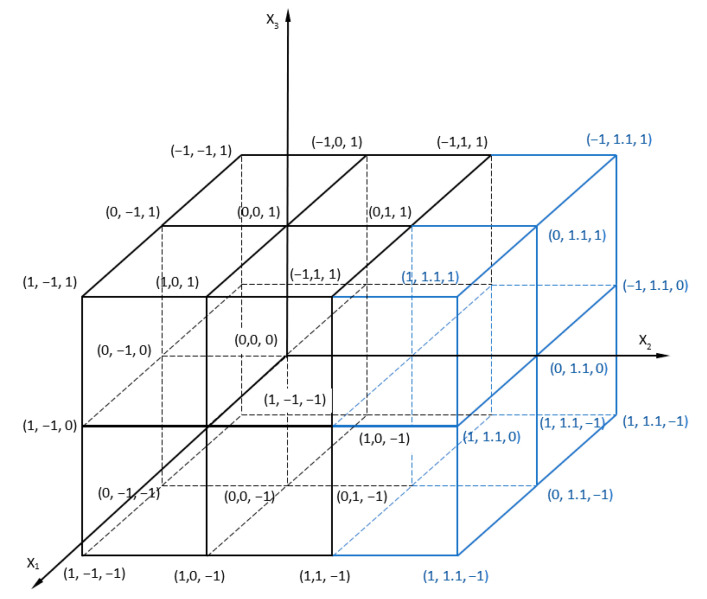
Graphical representation of the research plan.

The selection of the parameter range was carried out on the basis of preliminary tests [19]. On their basis, it was found that the welding time in the range of 1–1.5 s does not significantly affect the LC of the obtained joints. Increasing the welding time to 3.5 s allows for welds with fewer defects. However, further extension of time results in a significant reduction in the LC of the joints. Preliminary tests have also showed that increasing the tool depth leads to a gradual increase in the load capacity of the joints, which gradually decreases after reaching the maximum value. A simultaneous increase in the rotational speed to 2800 rpm and the welding time results in an increase in the load capacity of the joint. A further increase in rotational speed above the value 2800 rpm led to a weakening of the connection. Taking these conditions into account, the following parameters were determined: rotational speed of the tool *x*_1_ in the range of 2000 to 2800 rpm, the tool depth *x*_2_ in the range of 1.3 mm to 1.9 mm, and the welding time *x*_3_ (1.5–3.5 s). The RFSSW process was tested according to the three-factor three-level full factorial statistical completed plan PS/DC 3^3^. In order to more fully analyze the effect of tool depth on weld properties, the plan was extended by one level of the input factor *x*_2_ (Figure 3).

The joint load capacity in the static shear test was performed on the ZWICK Z100 test machine, with a jaw feed rate of 5 mm/min. A special holder was constructed to carry out the tests, reflecting the actual working conditions of the joint.

## 3. Results and Discussion

### 3.1. Experimental Results

In the initial stage of the investigation, the focus was on analyzing the impact of the depth of the plunge of the tool on the LC of the connection. The welds were made at a tool depth of *x*_2_ = 1.3 mm + *i* (*i* = 0.2, 0.4, 0.6 mm) and at rotational speeds in the range of 2000 to 2800 rpm.

**Figure 4:**
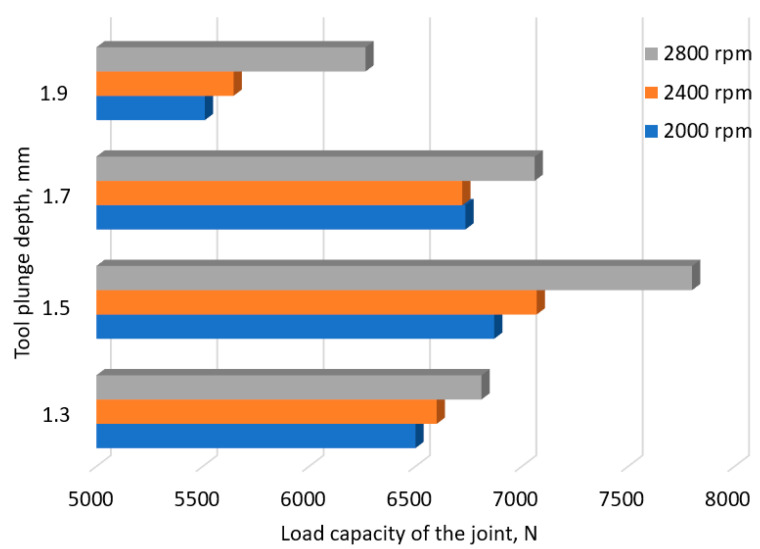
The force transmitted through the weld at different tool depths.

Analysis of the graph presented in Figure 4 indicates that an increase in rotational speed causes an increase in the LC of the connection, reaching its maximum value at 2800 rpm. The joint LC increases with the depth of the tool, so the maximum is obtained for the value *x*_2_ = 1.5 mm. Once this value is exceeded, all features tend to decrease in joint strength as the tool depth increases.

**Figure 5:**
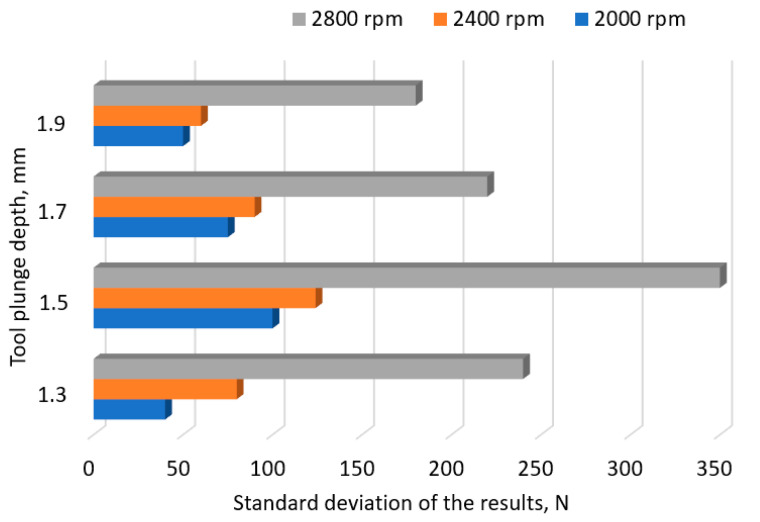
Standard deviation value at different tool plunge depths.

**Figure 6:**
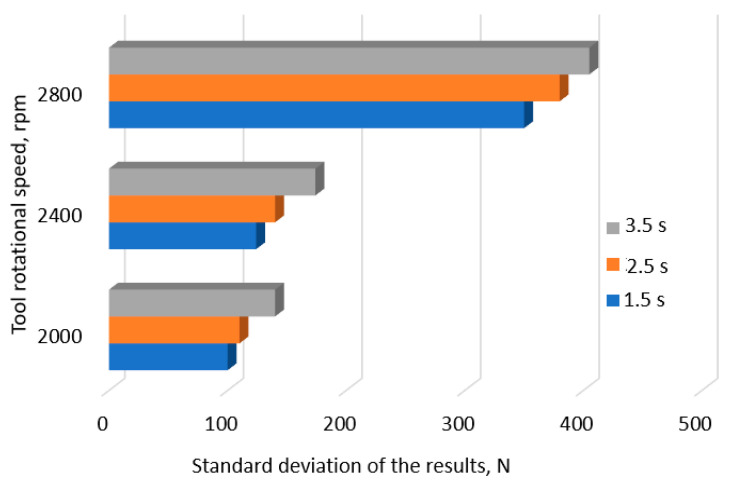
The force transmitted by the joint when the tool is recessed *x*_3_ = 1.5 s.

In aircraft structures, it is necessary to ensure that the LC of joints is distributed within a very narrow range. A small value of the standard deviation (SD) of the joint LC increases the quality of the process, which translates into the reliability of the joint. The tests carried out showed that the value of the SD depends on both the depth of the tool and the rotational speed. The highest values of the standard deviation were observed at the tool rotational speed of 2800 rpm (Figure 5) and at the pin working depth of 1.5 mm, i.e., the depth allowing the highest LC of the joint. In the second stage of the research, the impact of the tool rotational speed and welding time on the LC of the joints was analyzed. Figure 6 shows the results obtained with a tool depth of *x*_2_ = 1.5 mm.

Analysis showed that for the connection times *x*_3_ = 1.5 s and 2.5 s, increasing the rotational speed of the tool increases the LC of the joint. For time *x*_3_ =1.5 s, changing the rotational speed from 2000 rpm to 2800 rpm increases the LC by 17.9%, while for time *t* = 2.5 s, the LC is increased by 11.5%. In the case of welds made at time *x*_3_ = 3.5 s, it can be seen that increasing the rotational speed of the tool from 2000 to 2400 rpm increases the LC of the joint from 7000 N to 7587 N, that is, 8.3%. As a result of a further increase in the tool rotational speed by 16.6%, a 7.26% decrease in joint strength was observed, resulting from an excessive increase in joint temperature. The increase in temperature resulted in a deterioration of the tool’s operating conditions, leading to adhesive bonding of the material and an increase in its wear. The increased temperature of the joint caused overheating of the lower, thinner sheet, resulting in a significant increase in the dispersion of joint strength, reducing the quality of the process.

An increase in the tool rotational speed at a tool depth of *x*_2_ = 1.5 mm and a welding time of *x*_3_ = 2.5 s also leads to an increase in the LC of the joint. This is evidenced by the value of the linear correlation coefficient, which was *r* = 0.95. As the speed increases, the value of the SD of the result spread also increases, reducing the quality of the process (*r* = 0.90). With a welding time of *x*_3_ = 3.5 s, the LC of the connection increases with the increase in rotational speed. It reaches its maximum at a speed of *x*_2_ = 2400 rpm and then decreases. However, regardless of the adopted welding time, an increase in rotational speed leads to an increase in the SD. For the welding time *x*_3_ = 2.5 s, the value of the linear correlation coefficient was *r* = 0.91, while for the time *x*_3_ = 2.5 s, the value was *r* = 0.92. A similar tendency can be observed with a tool depth of *x*_2_ = 1.7 mm and a welding time of *x*_3_ = 1.5 s. An increase in rotational speed causes an increase in the LC of the joint and the SD. The correlation coefficient was *r* = 0.84 and *r* = 0.91, respectively. However, with a welding time of *x*_3_ = 3.5 s, an increase in the tool rotational speed leads to a decrease in the LC of the joint (*r* = −0.94). In the case of SD, a minimum value of 105 N was observed at a speed of *x*_2_ = 2400 rpm. An increase in the tool depth leads to an increase in the LC of the connection, reaching its maximum value at a tool depth of 93% of the thickness of the upper sheet. In this respect, a significant increase in the SD value was also observed.

**Figure 7:**
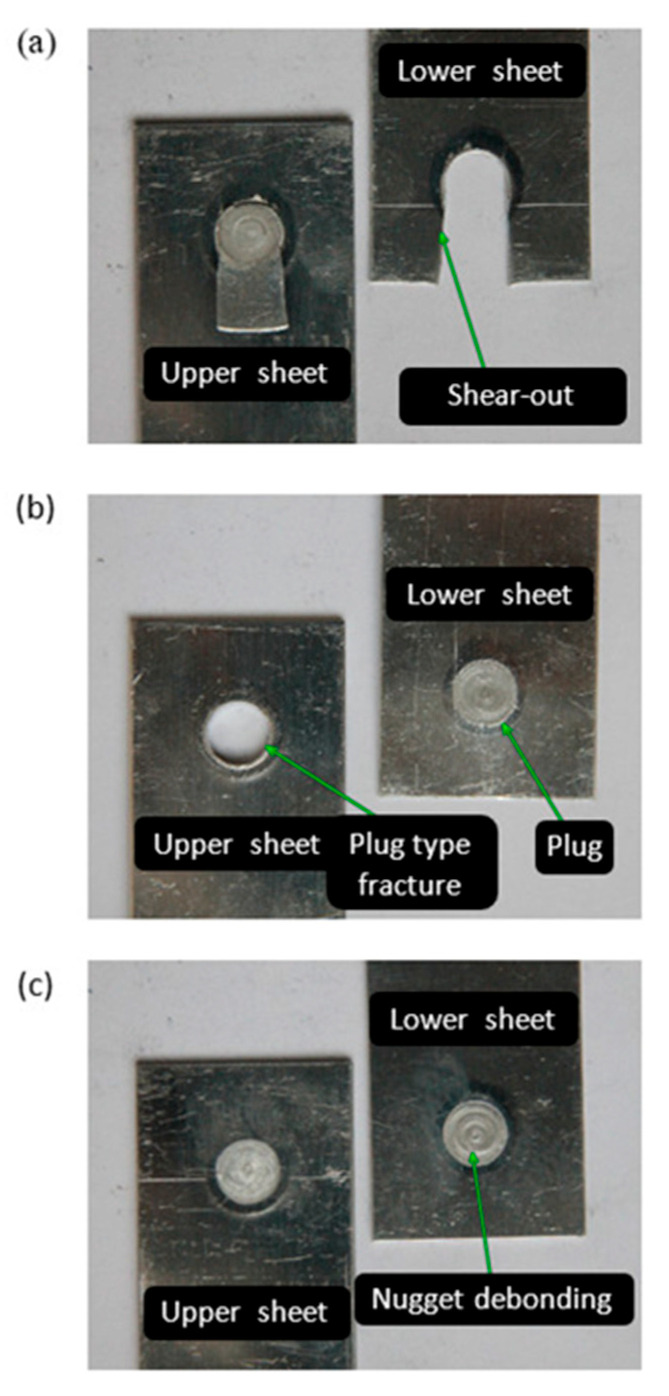
Failure modes after tensile/shear tests: shear-out failure (**a**), plug-type fracture (**b**), nugget debonding (**c**).

Three types of sample failures were observed during the static tensile/shear test of the joints. If a rotational speed of 2800 rpm is used and the welding time is 1.5 s and 2.5 s, type I failure of the samples was observed, characterized by the separation of fragments of the lower sheet (Figure 7a). The second type of damage was observed (Figure 7b) in the case of a low tool rotational speed (*x*_1_ = 2000 rpm), short welding time (1.5 s), and larger tool depth (1.7 mm and 1.9 mm). Reducing the tool depth (1.3 mm) led to type III failure of the sheets, characterized by a failure plane corresponding to the cross-section of the joint (Figure 7c).

In both cases, the joints made with the given parameters were characterized by the lowest LC due to the existence of a structural notch on the circumference of the weld. The presence of a distinct structural notch indicates that the sleeve penetrates too quickly into the material, which, instead of plasticizing the material, causes partial punching, while the temperature that arises later in the process leads to the recrystallization process, causing only partial reconstruction of the material. The resulting notch reduces the LC of the joints.

### 3.2. Mathematical Model

Ensuring the required quality capability of the process requires the determination of regression functions describing the joint strengths *L*(*x*) and the process dispersion *σ_L_*(*x*). The form of a third-degree polynomial was adopted to describe the process, taking into account the interactions between the parameters:

(1) $$W\left(x\right)=b_{0}+\sum_{i=1}^{S}b_{i}^{\left(1\right)}x_{i}+\sum_{\begin{array}{l} i,j=1\\ i<j \end{array}}^{S}b_{ij}^{\left(1\right)}x_{i}x_{i}+\sum_{\begin{array}{l} i,j,\ldots ,l,n=1\\ i<j,\ldots ,l<n \end{array}}^{S}b_{ij\ldots \ln}^{\left(1\right)}x_{i}x_{i}\ldots x_{l}x_{n}+\sum_{\begin{array}{l} i,j=1\\ i\neq j \end{array}}^{S}b_{ij}^{\left(2\right)}x_{i}^{2}x_{j}+\sum_{i=1}^{S}b_{ii\ldots m}^{\left(m\right)}x_{i}^{m}$$

in which there are L unknown coefficients $b_{0}$,$\;b_{i}^{\left(1\right)}$, $b_{ij}^{\left(1\right)}$, $b_{ij\ldots ln}^{\left(1\right)}$,$\;b_{ij}^{\left(2\right)}$,${\;b}_{ii\ldots m}^{\left(m\right)}$, with i, j, …, *n* = 1, …, S variables.

The assessment of compliance of the test results with the modeling results was carried out on the basis of the Fisher–Senecor statistic. For this purpose, the adequacy variance was determined (2):

(2) $$S_{ad}^{2}=\frac{r\;\sum_{i=1}^{N}{\left(y_{i}-\bar{y_{i}}\right)}^{2}}{N-k-1}$$

where: $y_{i}$—value determined based on the model, $\bar{y_{i}}$—average measurement results in the i-th trial, k—the number of the regression equation (without the intercept) after rejection of irrelevant terms, r—the number of repetitions, and N—number of experiments.

Then, the determined value of the test factor F (3) is as follows:

(3) $$F=\frac{S_{ad}^{2}\left(y\right)}{S^{2}\left(y\right)}$$

The obtained value was compared with the critical value determined from statistical tables, obtaining adequate regression Equations *L*(*x*) (4) and *σ_L_*(*x*) (5):

(4) $$L\left(x\right)=10,878.1-{18.751x}_{1}-0.0054x_{1}^{2}+2.34\cdot 10^{-6}x_{1}^{3}+{9753.3x}_{2}+{28.62x}_{1}x_{2}\;-0.004x_{1}^{2}x_{2}-14,944.4x_{2}^{2}-2.87x_{1}x_{2}^{2}+1921x_{2}^{3}-{4329.6x}_{3}+{8.87x}_{1}x_{3}-0.0019x_{1}^{2}x_{3}\;-3808.3x_{2}x_{3}+1187.5x_{2}^{2}x_{3}-1153.15x_{3}^{2}+164.087x_{3}^{3}$$

The maximum error value between the research and modeling results did not exceed 6.13%, while the mean square error was 2.58%.

The adequate regression function describing process variability (5) is simpler. The mean square error between the modeling and test results was 10.60%, while the maximum error did not exceed 14.51%.

(5) $$\sigma_{L}\left(x\right)=-8585.36-{1.0813x}_{1}+{18,367.7x}_{2}-{63.958x}_{3}+{0.1364x}_{1}x_{2}+{0.0164x}_{1}x_{3}\;+0.0000838x_{1}^{2}-10739.6x_{2}^{2}+9.791x_{2}^{2}x_{3}+6.304\cdot 10^{-8}x_{1}^{3}+2094.91x_{2}^{3}\;\;\;\;\;\;\;\;\;\;\;\;\;\;\;\;\;\;\;\;\;\;$$

### 3.3. Artificial Neural Networks Modeling

Multi-Layer Neural Networks (MLNNs) used in analyses consists of three layers: an input layer, a hidden layer, and an output layer. The input layer contained three neurons corresponding to the analyzed input signals. In turn, the output neuron was responsible for determining the response of the feedforward neural network. Each neuron in a specific layer is connected to every neuron in the next layer. Neurons sum up the input signals, taking into account the weights. When the calculated sum value exceeds the threshold value, the neuron is excited. The signal representing the total activation of the neuron is, in turn, transformed by the activation function of the neuron. The value calculated by the activation function is the output signal of the neuron. In the analyzed unidirectional MLNNs, there is no feedback. A single signal passes through each neuron exactly once in the cycle of training and predicting the output signal. Neurons in individual layers are connected to each other, and the weights of these connections are modified during the training process. Data analysis using Multi-Layer Neural Networks (MLNNs) makes it possible to analyze often complex relationships between explanatory variables and an output variable. In this article, the MLNNs were built using the Statistica program. The objective of the study was to determine the impact of welding parameters on the LC of joints Fc and the SD of the LC of joints sc. The duration of welding, plunge depth, and tool rotational speed were considered as input parameters in the neural network. The parameters expected at the network output were the SD of load capacity and the load capacity of joints. Multi-layer neural networks with one hidden layer were considered. Hertz et al. [20] found that a three-layer network is sufficient to model any nonlinear function. The Statistica program allows for the automatic generation of neural network architectures based on the entered experimental data. This article tested the regression capabilities of the neural network. Therefore, the experimental dataset has been divided into a learning set and a validation set in the proportion of 85% and 15%, respectively. A back propagation algorithm was used to train the network. The Statistica program automatically carries out the learning process. Min–max normalization (Equation (6)) was used to linearly transform the original data to the range (0, 1):

(6) $$x_{scaled}=\frac{x-x_{min}}{x_{max}-x_{min}}$$

where *x* is the normalized parameter, and *x*_min_ and *x*_max_ are the minimum and maximum values of the normalised parameter, respectively.

The prediction quality of the MLNNs was assessed based on the root mean square (RMS) error [21]. Analyses were performed for networks with a different number of neurons in the hidden layer (the structure of the input and output layers was determined based on the number of input and output parameters). The value of the RMS for the training set was adopted as the network quality criterion. In addition to this parameter, the most common indicators of the quality of the network forecast are the Pearson’s correlation and the standard deviation ratio (SDR). When evaluating a regression model, it is well-known that the higher the value of the Pearson’s correlation for the learning set, the higher the quality of the prediction. At the same time, an SDR value lower than approximately 0.2 means very good generalization ability of the neural network. The Statistica program automatically performed analyses with various network architectures and determined the network that provided the lowest RMS error value. Regression statistics of the analyzed neural networks used for predicting the value of the LC of the joint and the SD of joint capacity are listed in Table 1 and Table 2, respectively.

**Figure 8:**
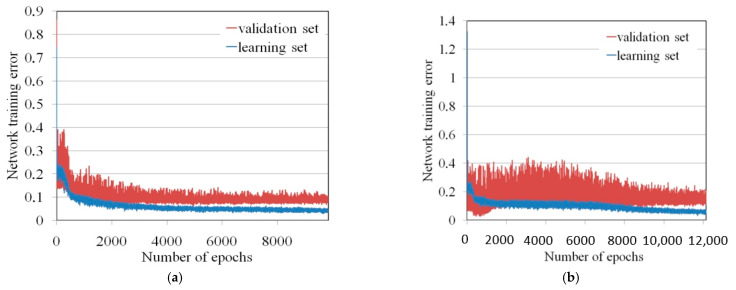
Variation of the network error during training (**a**) the MLNN 3-16-1 and (**b**) MLNN 3-15-1.

Initially, the MLNN errors were large for randomly selected values of the neuron activation function. During the learning process, the error of the training and validation sets decreased (Figure 8). The error value and the spread of error values for the training set were smaller because this set contained a larger amount of data.

Table 3 presents the results of the sensitivity analysis of the obtained MLNN models. The ‘rank’ row specifies the importance of a given variable in the neural model (1—the most important, 3—the least important). The ‘error’ row contains the total network error resulting from removing the variable corresponding to a given column from the input parameter (*x*_1_*, x*_2_ or *x*_3_). The ‘rank’ row contains the quotient value:

(7) $$rank=\frac{error\;obtained\;after\;deleting\;the\;specific\;variable}{error\;of\;the\;network\;taking\;into\;account\;all\;input\;variables}$$

Tool plunge depth is the parameter with the greatest information value at the input of the 3-15-1 neural network predicting the value of the LC of RFSSW joints. The network error resulting from removing this variable is the largest (858.631). The least important parameter in terms of significance is the duration of welding. In MLNN 3-16-1 predicting the SD of load capacity output, tool rotational speed is the most sensitive. Therefore, it can be deduced that the rotational speed determines the spread of the LC of RFSSW joints. As in the case of the 3-15-1 MLNN network, welding time is the least important parameter in terms of the significant impact on the SD of load capacity.

### 3.4. Comparative Analysis of Model Results

**Figure 9:**
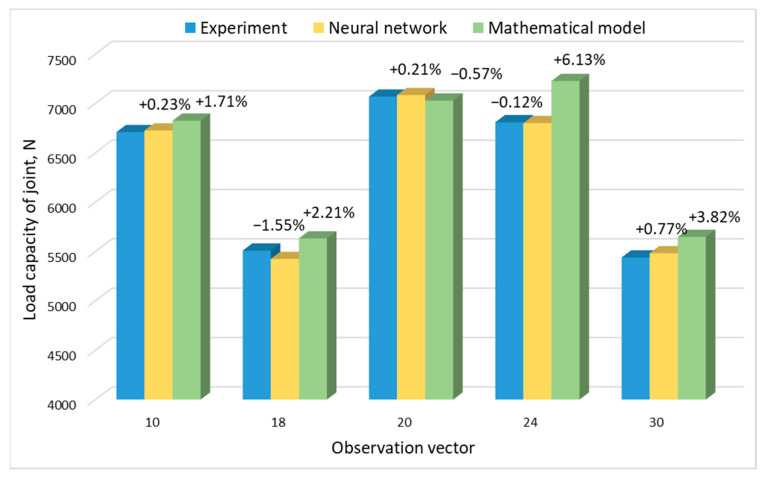
Comparison of the results of experimental studies of joint load capacity with the results of the mathematical model and with the use of a neural network.

**Figure 10:**
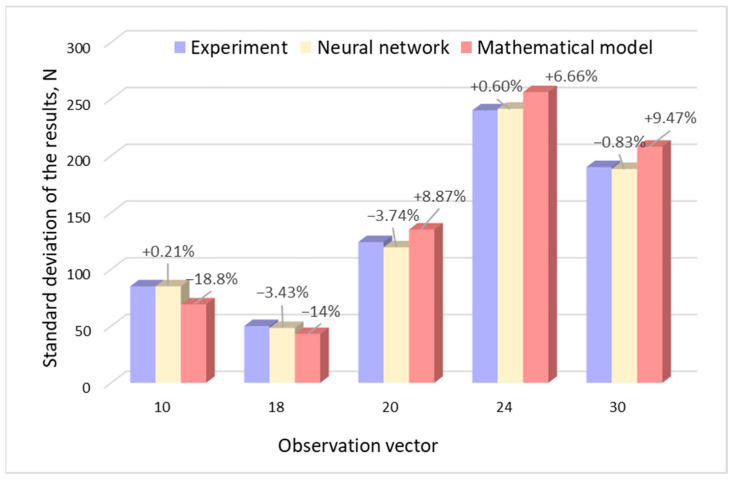
Comparison of experimental results of the SD of the joint with the results of the mathematical model and with the use of a neural network.

The neural network prediction test was conducted on the basis of four input vectors (parameters), with which the joints were made and their load capacity was tested. Comparison of the results of the neural network, experimental research, and the results obtained on the basis of mathematical modeling are shown in Figure 9 and Figure 10.

In both the case of the joint’s load capacity and the SD of joint capacity, the results obtained by the neural network were characterized by a smaller error value. In the case of load capacity, the maximum error did not exceed 1.55%, while in the case of prediction, the standard deviation was 3.74%.

Neural networks have many advantages over classical mathematical modeling that can be used in practice. They do not require complex and time-consuming calculations necessary to develop a regression equation. The accuracy of the obtained mathematical model depends largely on the knowledge and experience of the person conducting the research. In the case of neural networks, the programmer’s role is limited to designing the network structure that will be best suited to solving a given problem and then skillfully managing the network training process. Neural networks also make it possible to take into account a much larger number of factors influencing the process, which significantly increases their forecasting accuracy. However, they have their limitations, especially when optimization of process parameters is required. In such cases, a more advantageous solution is to use a classical mathematical model.

**Figure 11:**
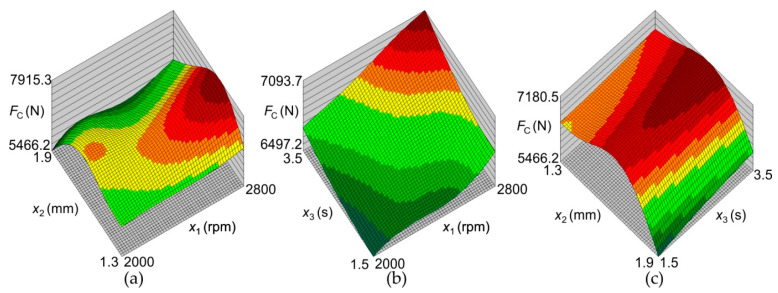
Response surfaces presented the effect of welding parameters on the joint load capacity *F*_c_: (**a**) *F*_c_ = f(*x*_1_, *x*_2_), (**b**) *F*_c_ = f(*x*_1_, *x*_3_), and (**c**) *F*_c_ = f(*x*_2_, *x*_3_).

**Figure 12:**
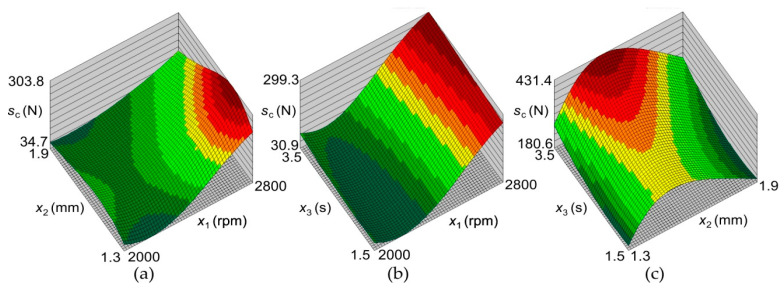
Response surfaces presented the effect of welding parameters on the SD of the LC sc: (**a**) sc = f(*x*_1_, *x*_2_), (**b**) sc = f(*x*_1_, *x*_3_), and (**c**) sc = f(*x*_2_, *x*_3_).

**Figure 13:**
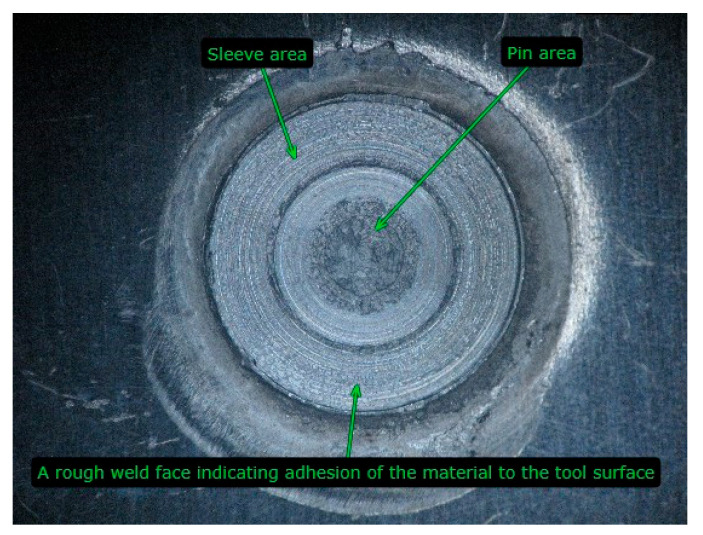
Surface of the RFSSW joint.

Figure 11a shows an increase in the joint LC with an increase in the tool rotational speed (changes in colour: green the lower value and red the higher value). The highest load capacity occurs at the tool rotational speed of 2800 rpm and the tool plunge depth of 1.5 mm. The response surface for the load capacity is very similar to that for the SD of joint capacity (Figure 12a). At a tool rotational speed of 2000 rpm, the SD of the LC of the joints is similar throughout the range of tool plunge depths analyzed. The large SD for joints obtained at high tool rotational speeds results from high friction, which causes an increase in temperature and a tendency for the BM to adhere to the tool surface, and, consequently, an increase in the unevenness of the weld surface (Figure 13) [22,23]. These welding conditions ensure the highest LC of the joint, and at the same time, the highest SD of the load capacity occurs (Figure 11a and Figure 12a). The LC of the joints increases with a simultaneous increase in the tool rotational speed and the duration of welding (Figure 11b). There is a clear difference in the response surface for the load capacity (Figure 11b) and the SD of joint capacity (Figure 12b). The load capacity value (Figure 11c) is strongly correlated with its standard deviation value (Figure 12c). The range of highest load capacity with SD of load capacity is for tool plunge depths between 1.5 and 1.7 mm (changes in colour: green the lower value and red the higher value). The increase in the duration of welding at a specific plunge depth has a slight effect on the increase in the LC and the SD.

**Figure 14:**
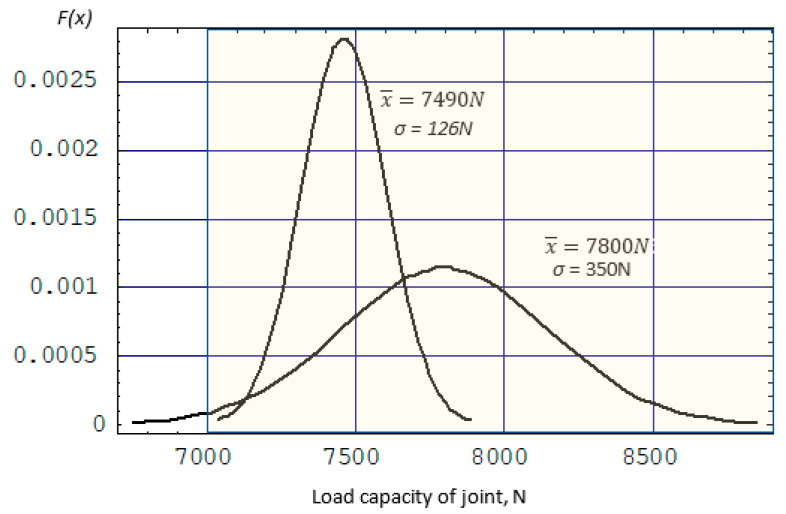
The probability density function.

To ensure an appropriate level of structural reliability, connections made using the RFSSW method should be characterized by a high load capacity with relatively small spread. Due to this, the process will be statistically controllable (predictable in its behavior), and it will be possible to distinguish disturbances that occur in the process (special causes) from the natural variability of the process (normal causes). In the case of the tested joints, the highest value of the LC ($\bar{x}$ = 7800 N) was obtained at a rotational speed of the tool of *x*_1_ = 2800 rpm, a tool depth of *x*_2_ = 1.5 mm, and a welding time of 1.5 s, with the SD of the process dispersion equal to σ = 350 N (Figure 14). High structural reliability requires the highest possible connection load capacity. Therefore, structural connections are characterized by a one-sided interval (DWG). Therefore, the probability of obtaining correct welds can be determined by numerically calculating the integral [24,25] (8):

(8) $$P\left(X\geq DWG\right)=\int_{DWG}^{\infty }\frac{1}{\sqrt{2\pi }\sigma }e^{\frac{-{\left(x_{śr}-x\right)}^{2}}{2\sigma^{2}}}dx$$

The probability of obtaining welds with a load capacity greater than 7000 N with an average value of $\bar{x}$ = 7800 N and a standard deviation σ = 350 N is 0.9888. This corresponds to a process quality capability of C_p_ = 0.84 and a process failure rate of 1.12%.

**Figure 15:**
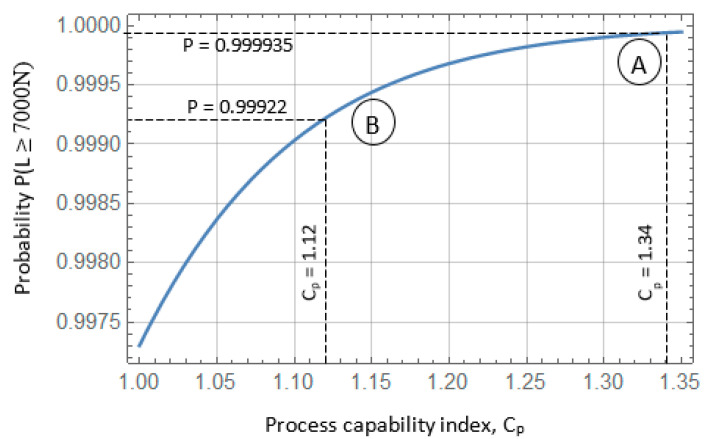
Dependence of the C_p_ index value on the probability of obtaining the connection load capacity L ≥ 7000 N.

Analysis of the response surfaces obtained from the model based on the neural network (Figure 11 and Figure 12) indicates that as the rotational speed of the tool decreases, not only does the value of the joint LC decrease, but also the standard deviation. The LC of the joint can be increased by increasing the welding time. With a tool rotational speed of 2256 rpm, a tool depth of 1.55 mm, and a welding time of 3 s, an average load capacity of joint $\bar{x}$ = 7490 N can be obtained with a standard deviation of σ = 126 N. With these parameters, the probability of obtaining welds with a load capacity greater than 7000 N is 0.999935. The process defect in this case is much lower and reaches 0.0065%, which corresponds to the qualitative efficiency of the process C_p_ = 1.34 (Figure 15, point (A)).

It should be noted that the LC and SD of connections determined on the basis of a classic mathematical model are subject to a much higher error value, which translates into the value of the process quality capability coefficient. For the presented process parameters, the mathematical model makes it possible to determine the SD with an error of 12.6% (142 N). As a result, the determined value of the process quality capability index Cp = 1.12 (defectiveness level 0.078%) is significantly underestimated (Figure 15, point (B)). This may lead to wrong decisions involving the rejection of the most favorable process parameters and the adoption of inappropriate connections, ensuring lower reliability.

In order to increase the process capability, it is necessary to carry out a multi-criteria optimization process of RSSW parameters based on a classic mathematical model. In such a case, it would be beneficial to use a directed optimization model to solve the problem, which allows for the selection of process parameters that ensure a compromise between load capacity, standard deviation, and weld time. The results obtained on this basis should be verified with a neural model and used in the next optimization step.

## 4. Conclusions

The article presents research on the use of artificial neural networks to predict the maximum force transmitted through the joint and its SD. The results obtained provide optimistic premises regarding the possibility of using them to describe the RFSSW process. As a result of numerical experiments, a neural network structure was selected that correctly predicts the load capacity of the joint. The average prediction error for this network did not exceed 0.72% for the maximum force transmitted through the joint and 2.2% for the standard deviation. The mathematical model is characterized by much higher error values. In the case of force transmitted through the joint, the average error value is more than twice that of the joint and amounts to 3.26%. For the standard deviation, this value is 14.45%.

The following conclusions were drawn:

1. The rotational speed of the plasticizing pin determines the appropriate temperature in the weld area, allowing proper plasticization and mixing of the material. Too high a tool rotational speed adversely affects the microstructure of the weld. As the rotational speed increases, the standard deviation of the joint load capacity also increases, leading to heterogeneity in the joint performance properties.
2. In the case of lap joints, the highest joint load capacity can be obtained with a tool recess of 93% of the thickness of the cover plate. In this case, the tool does not penetrate the material of the lower sheet. This creates an adhesive connection. Increasing the depth of the tool leads to a weakening of the thin bottom sheet, which results in a reduction in the LC of the connection.
3. Increasing joint load capacity can be achieved by increasing welding time (3.5 s), using a depth of 1.5 mm (93%) and a tool rotation speed of 2250 rpm. The process of welding with such parameters is characterized by smaller scatter. In this way, greater uniformity can be achieved in the performance properties of joints, which may contribute to greater structural reliability.
4. Ensuring high quality in the aircraft structure assembly process requires precise determination of welding parameters. Such conditions are met by the neural model, which is characterized by much greater accuracy in predicting the load capacity of joints and standard deviation than the classical model. This also translates into the precision of estimating the value of process quality capability indicators required by the AS9100 standard, which is intended for companies specializing in aviation production.
5. The use of neural networks enables precise determination of welding process parameters, ensuring maximum LC of the joints with a minimum SD of the process spread. However, this does not ensure their optimization. Based on the research carried out on the welding process of thin-walled sheets, it is indicated that in industrial practice, maximum rotational speeds should not be used when selecting parameters. The required LC of the joints should be obtained by increasing the welding time. This allows for more uniform welds with fewer defects.

Developing an accurate model requires conducting many tests, which are often time-consuming and, due to the materials used in the aviation industry, also expensive. Therefore, taking this aspect into account, it seems that the classic method of mathematical modeling may be used more often in less demanding industries (e.g., automotive). Its results are subject to a higher error value, but it requires much less work.

The conducted tests indicate that an important issue that could be developed in subsequent research is the issue of tool wear. The plasticized metal contaminates the tool with adhesion, contributing to its faster wear and thus changing the tool geometry. The induced change in tool geometry leads, in turn, to the formation of structural defects in the weld, resulting in a reduction in the load capacity of the joint. This requires the introduction of protective coatings on the working elements of the tool, followed by research on the impact of protective coatings on the amount of tool wear and weld properties. The introduction of protective coatings additionally enables testing of the process with an increased cooling rate using liquids.

## Figures and Tables

**Table 1:** Network regression statistics for predicting load capacity of RFSSW joints.

MLNN Structure	RMS Error	Error of SD	Absolute Error Mean	SDR	Pearson’s Correlation
3-15-1	82.04	83.375	64.989	0.129	0.992

**Table 2:** Network regression statistics for predicting SD of load capacity of RFSSW joints.

MLNN Structure	RMS Error	Error of SD	Absolute Error Mean	SDR	Pearson’s Correlation
3-16-1	10.69	9.474	8.376	0.092	0.995

**Table 3:** Results of sensitivity analysis.

Network Architecture	Parameter	*x*_1_	*x*_2_	*x*_3_
3-15-1	rank	2	1	3
	error	375.029	858.631	258.425
	ratio	4.571	10.466	3.150
3-16-1	rank	1	2	3
	error	101.355	71.451	29.393
	ratio	9.484	6.686	2.750

## Data Availability

The data presented in this study are available upon request from the corresponding author.
